# Do psycho-behavioural interventions improve mental and physical health in chronic kidney disease? A systematic review of randomised controlled trials

**DOI:** 10.1007/s40620-025-02372-9

**Published:** 2025-09-10

**Authors:** Pooja Schmill, Natasha Seaton, Sharlene Greenwood, Joanna L. Hudson, Emily McBride, Sam Norton, Joseph Chilcot

**Affiliations:** 1 Department of Psychology, Institute of Psychiatry, Psychology & Neuroscience, Health Psychology Section, King’s College London, 5th Floor Bermondsey Wing, Guy’s Campus, London Bridge, London, SE1 9RT UK; 2 Institute of Psychiatry, National Institute for Health and Care Research (NIHR) Maudsley Biomedical Research Centre (BRC), Psychology & Neuroscience, London, UK; 3 King’s College Hospital, NHS Foundation Trust, London, UK; 4 Department of Inflammation Biology, Faculty of Life Sciences & Medicine, King’s College London, London, UK

**Keywords:** Chronic kidney disease, Psychological interventions, Behavioural therapy, Mental health, Randomised controlled trials

## Abstract

**Background:**

Depression and anxiety are common in chronic kidney disease (CKD) and worsen clinical outcomes. Psycho-behavioural interventions offer a promising, non-pharmacological approach. However, most evidence comes from people with kidney failure with distinct treatment needs, limiting relevance to earlier stages of CKD, where timely support may enhance self-management and slow progression. This systematic review evaluates the effectiveness of psycho-behavioural interventions in adults with CKD without dialysis or transplantation.

**Methods:**

We searched MEDLINE, EMBASE, PsycINFO, Cochrane Central, and Web of Science (inception–March 2025) for randomised controlled trials (RCTs) testing psycho-behavioural interventions in adults with CKD (not on kidney replacement therapy), with depression and/or anxiety as primary or secondary outcomes. Risk of bias (RoB-2) and certainty of evidence were assessed. Given methodological heterogeneity across studies, vote counting by effect size and narrative synthesis were applied. PROSPERO: CRD42024515733.

**Results:**

Five RCTs (*N* = 631) met the inclusion criteria, evaluating cognitive behavioural therapy, self-efficacy training, mindfulness-based stress reduction, and physical activity, delivered digitally, by phone, or in person. Moderate-certainty evidence showed consistent improvements (100% positive) in self-efficacy and physical function. Low-certainty evidence indicated 100% positive effects on self-management, while findings for depression were mixed (67% positive), with one study reporting worsening symptoms. Evidence for anxiety, fatigue, quality of life, and kidney function was inconclusive due to high inconsistency and imprecision.

**Conclusion:**

Psycho-behavioural interventions may enhance self-efficacy, self-management, and physical function in CKD. However, evidence for mental health and kidney outcomes remains limited. Robust, long-term RCTs with tailored, multi-component approaches are needed to support integration into kidney care.

**Graphical Abstract:**

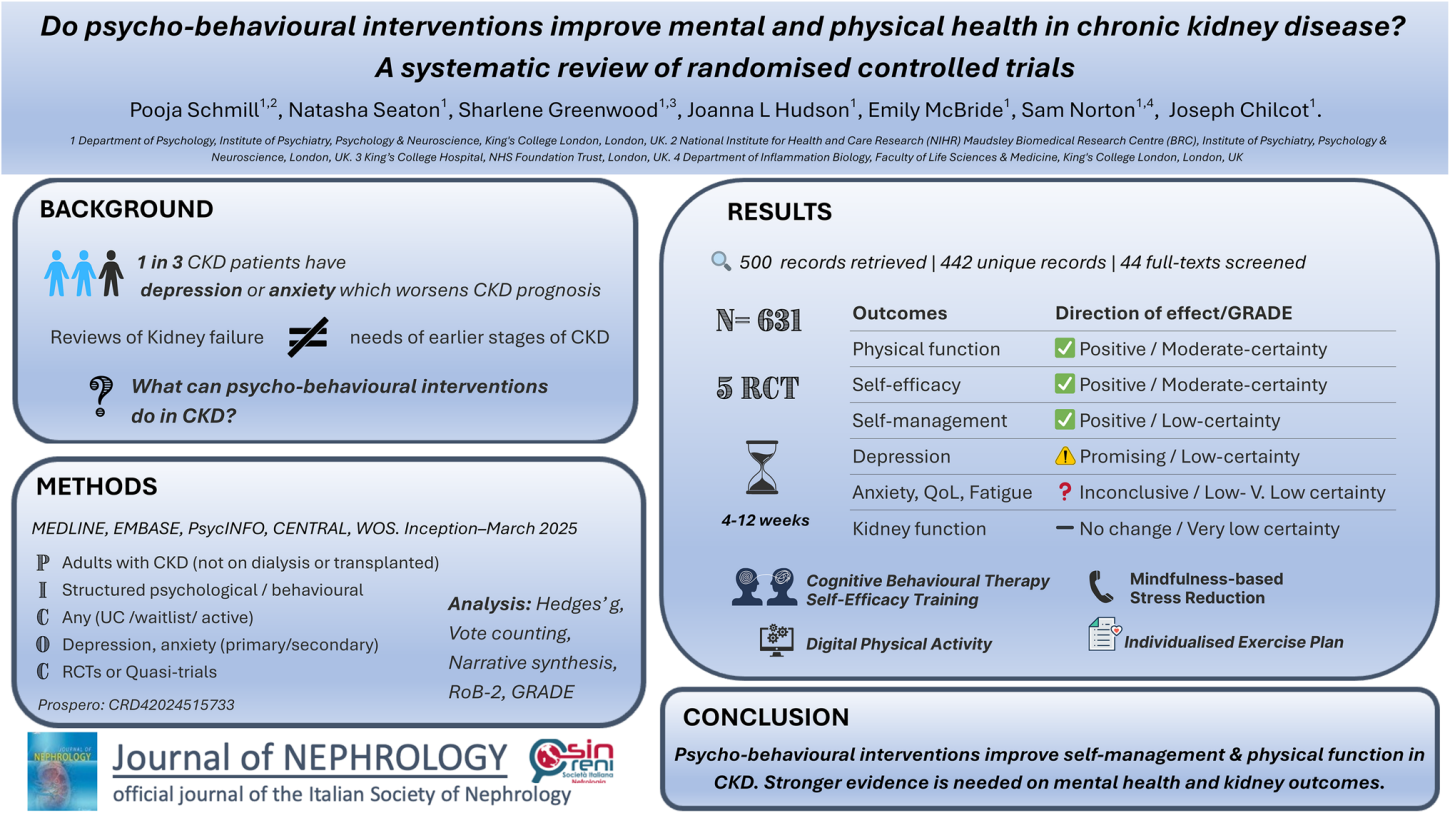

## Introduction

Chronic kidney disease (CKD) affects 10–15% of adults globally and represents a growing public health concern [1]. Defined by an estimated glomerular filtration rate (eGFR) < 60 mL/min/1.73 m^2^ or markers of persistent kidney damage for over three months, CKD progresses through five stages culminating in kidney failure, where kidney replacement therapy (KRT) becomes necessary [2].

Depression and anxiety are highly prevalent in CKD, exceeding rates in both the general population and other chronic diseases [3, 4]. Meta-analyses report clinically significant depression in 26.5% and anxiety disorders in approximately 19% of CKD patients [5, 6]. Psychological distress is multifactorial, driven by disease chronicity, symptom burden, and lifestyle restrictions [7]. Crucially, depression and anxiety in CKD are associated with adverse clinical outcomes, including faster disease progression, increased hospitalisation, reduced quality of life (QoL), and elevated risk of all-cause mortality [8–11].

Psychological interventions offer a safer, person-centred alternative to pharmacotherapy for managing mental distress in CKD, given the risk of adverse effects, such as gastrointestinal bleeding and altered drug metabolism, associated with psychotropic medications in this population [12, 13]. Meta-analyses report moderate-to-large effects of psychotherapies in reducing depression (Hedges’ *g* = 0.71; 95% CI [0.59, 0.82]) and anxiety (Hedges’ *g* = 0.84; 95% CI [0.71, 0.97]) in the general population [14], with small-to-moderate effects observed for cognitive behavioural therapy (CBT) in chronic disease populations (depression: Hedges' *g* = 0.61; 95% CI [0.49, 0.72]; anxiety: Hedges’ *g* = 0.56; 95% CI [0.42, 0.70]) [15].

To date, most research on psychological intervention in CKD has concentrated on patients with kidney failure on KRT (dialysis or transplantation). For example, a Cochrane review of 33 randomised controlled trials (RCTs; *N* = 2056) involving dialysis patients found evidence supporting the effectiveness of Cognitive Behavioural Therapy (CBT; MD =  − 6.10; 95% CI [− 8.63, − 3.57]), exercise interventions (MD =  − 7.61; 95% CI [− 9.59, − 5.63]), and relaxation techniques (MD =  − 5.77; 95% CI [− 8.76, − 2.78]) in reducing depressive symptoms [16]. Similarly, a recent meta-analysis of 19 RCTs [17] reported significant reductions in depression scores measured using Beck Depression Inventory (BDI; WMD =  − 3.27; 95% CI [− 7.81, 1.27]), albeit with considerable heterogeneity (*I*^*2*^ = 95.1%). However, the focus on kidney failure populations limits generalisability, as kidney failure patients comprise only ~ 3.7% of the total CKD population [1]. Earlier stages of CKD differ significantly in symptom burden, self-management potential, and intervention receptivity, necessitating targeted research to guide care in these earlier phases [3].

Emerging evidence and patient consensus increasingly call for psychological care to be embedded earlier in the CKD care pathway [18]. The initial stages of the CKD journey represent a critical window for intervention, where preventing deterioration in mental health may mitigate downstream effects on disease progression, treatment adherence, and QoL. Depression demonstrates a dose-dependent relationship with worsening CKD outcomes [3]. Several biological and behavioural mechanisms underpin this relationship, including uraemic toxin accumulation, neuroinflammation, hypothalamic–pituitary–adrenal (HPA) axis dysregulation, poor treatment adherence, maladaptive coping behaviours, and diminished social support [3].

Despite this, there is a paucity of high-quality evidence evaluating psychological and behavioural interventions specifically in CKD populations not yet receiving KRT. Addressing this gap is crucial for developing preventative, scalable mental health strategies tailored to the unique needs of this population. Therefore, the objective of this systematic review was to evaluate the efficacy of psychological and behavioural interventions on depression and anxiety in CKD patients not on KRT. By restricting inclusion to RCTs, we sought to ensure methodological rigour and minimise bias inherent to non-randomised designs. Specifically, this review aimed to address the following research questions:

1. Are psychological and behavioural interventions effective in reducing depression and anxiety in CKD patients not on KRT?
2. Do these interventions improve secondary clinical outcomes, such as kidney function, hospitalisation rates, mortality, and quality of life?

This synthesis of high-quality evidence is intended to inform clinical guidelines and the design of future interventions, advancing integrated, whole-person care in CKD.

## Methods

This systematic review was prospectively registered (PROSPERO: CRD42024515733) and conducted according to Preferred Reporting Items for Systematic reviews and Meta-Analyses (PRISMA) 2020 guidelines [19] (See Supplementary materials – Table 1).

**Table 1:** PICOS eligibility criteria

	Inclusion criteria	Exclusion criteria
Population	Adults (≥ 18 years) with CKD, not on KRT (dialysis/transplant)	Non-CKD (acute kidney injury/disease), on KRT, animal studies
Intervention	Structured psychological and/or behavioural interventions	Unstructured interventions, studies not assessing depression/anxiety, drug interventions
Comparator	Any control group	No comparator
Outcomes	Standardised measures of depression and anxiety	No pre/post outcome measures
Study design	RCTs or quasi-RCTs	Non-randomised studies

*CKD* chronic kidney disease, *KRT* Kidney Replacement Therapy, *RCT* Randomised Controlled Trial, *Psycho-behavioural interventions* Structured approaches aimed at modifying dysfunctional thoughts, emotions, or behaviours—including cognitive therapies, CBT, IPT, MBCT, ACT, psychodynamic, supportive, and behavioural therapies—along with counselling and psycho-behavioural interventions (e.g., exercise therapy) beyond usual care or informational

### Eligibility criteria and study selection process

**Fig. 1:**
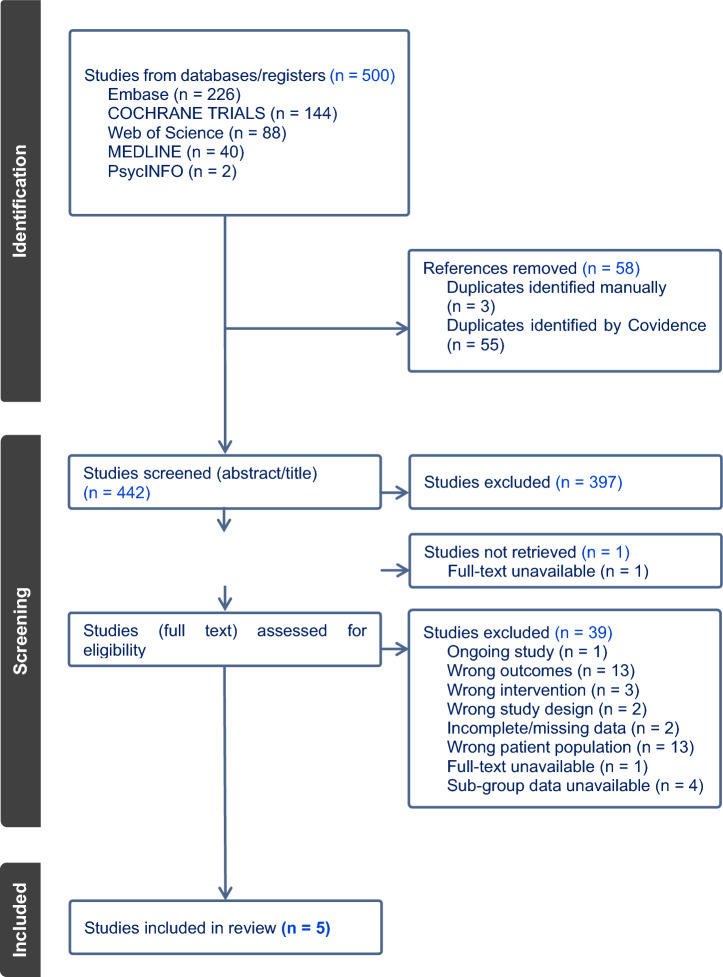
PRISMA flowchart attached separately

References were imported, screened, and managed using Covidence (Covidence—Better systematic review management) which is a review management software. Duplicate records were automatically removed by Covidence, and two reviewers (PS & NS) independently screened studies using the PICOS framework, resolving disagreements through consensus [20]. Eligible studies were RCTs or quasi-RCTs assessing depression or anxiety as a primary or secondary outcome in CKD adults not on KRT. Studies with ≥ 40% non-KRT CKD participants or available sub-group data were included to widen inclusion criteria whilst ensuring results reflected CKD populations. No sample size restrictions were applied. The study selection process was documented using a PRISMA flowchart (Fig. 1).

### Search strategy

A systematic search was conducted in MEDLINE (Ovid), EMBASE (Ovid), PsycINFO (Ovid), Cochrane CENTRAL, and Web of Science (inception–March 2025, with no language restrictions. Reference lists of included studies were hand-searched, and corresponding authors were contacted when full-texts were unavailable. The search strategy, combining MeSH terms and free-text keywords related to CKD, psychological interventions, and study design, was reviewed by a librarian and detailed in materials (See supplementary materials Table 2).

**Table 2:** Summary of Study Characteristics

Study (Year)	Country	Study Design	Sample (n)	Age Mean (SD) Intervention/ Control	% Male Intervention/ Control	% White Intervention/ Control	Intervention Type	Comparator	Duration (Intervention/ Follow-up)	Reported Outcomes	Inclusion Criteria
Greenwood (2024)	UK	RCT	340	53.9 (13.6)/53.8 (13.5)	55%/53%	73%/77%	Digital physical activity (Kidney Beam)	Wait-list control	12 weeks/12 weeks	Depression, anxiety, fatigue, Self-management, physical function, KDQoL, eGFR	Adults (≥ 18 yrs) with established CKD (including KRT), digital access, English-speaking
Gross (2017)	USA	RCT	63	52.6 (12.6)/54.6 (11.7)	30%/67%	70%/82%	Telephone MBSR (tMBSR)	Telephone support (attention control)	8 weeks/12 and 24 weeks	Anxiety, depression, fatigue, QoL	Adults (≥ 18 yrs) with CKD awaiting transplant, not on KRT, English-speaking
Picariello (2024)	UK	RCT	26	43.1 (10.4)/54.0 (10.3)	7.7%/23.1%	92.3%/92.3%	Digital CBT (COMPASS)	Standard charity support (SCS)	12 weeks/12 weeks	Depression, anxiety, QoL	Adults (≥ 18 yrs), PHQ-4 ≥ 3, self-reported LTC incl. CKD, digital literacy, English-speaking
Tang (2017)	China	RCT	90	46.3 (15.6)/43.9 (12.4)	66.7%/54.8%	Not reported	Individualised exercise program	Usual care	12 weeks/12 weeks	Depression, anxiety, Physical function, QoL, self-efficacy	Adults (18–70 yrs) with CKD stages 1–3, contemplation/ preparation stage of change, English-speaking
Wu (2018)	Taiwan	RCT	112	67.8 (9.4)/71.7 (12.7)	68.9%/5 6.7%	Not reported	Self-efficacy-based self-management	Usual care	4 weeks/4 weeks	Depression, anxiety, Self-efficacy, self-management, eGFR	Adults (≥ 20 yrs) with CKD stages 3b–5 > 3 months, Mandarin/ Taiwanese-speaking, attended ≥ 3 of 4 group sessions

### Data extraction

Data extraction was conducted by two independent reviewers (PS and NS) using standardised templates within Covidence, capturing study characteristics (author, year, country, design, sample size), participant demographics (age, sex, CKD stage), intervention details (mode, duration, frequency), comparator type, and primary/secondary outcomes. Extracted data were cross-verified, and discrepancies were resolved by consensus (PS & NS), where required. Intercoder reliability (Cohen’s *κ* = 0.66, 91.1% agreement) confirmed substantial agreement. Intervention data extraction by one author (PS) followed the template for intervention description and replication (TIDieR) to ensure standardised reporting [21] (Table 1).

### Risk of bias assessment

The Cochrane Risk of Bias tool for RCTs (RoB-2) was used to evaluate selection, performance, detection, attrition, and reporting bias [22]. Two independent reviewers (PS & NS) assessed risk, resolving discrepancies through consensus. Studies were rated as low, high, or unclear risk. Certainty of evidence was assessed by one reviewer (PS) using Grading of Recommendations Assessment, Development and Evaluation (GRADE) classifying evidence as high, moderate, low, or very low [23].

### Data synthesis

Given the heterogeneity in interventions and outcome measures, a meta-analysis was not feasible. Instead, Synthesis Without Meta-analysis (SWiM) was applied following Cochrane Handbook guidelines [20]. To assess intervention effects, we calculated Hedges’ g for all relevant outcomes based on author-reported means, standard deviations (SDs), and sample sizes (N). Effect sizes (i.e., Hedges’ g estimates) were reported with 95% confidence intervals (CIs) to facilitate the interpretation of the precision of treatment effect estimates alongside determining intervention effectiveness.

For primary outcomes (depression and anxiety), vote counting based on effect size direction was conducted, classifying intervention effects as positive (beneficial), unclear (inconclusive), or negative (harmful). This approach is recommended over vote counting by statistical significance, as it reduces bias associated with study sample size and p-value thresholds [20] providing a more reliable synthesis of evidence. To further account for precision, we incorporated 95% CIs, ensuring that the interpretation of effect direction considered potential random variation. Secondary mental and physical health-related outcomes (fatigue, self-management, self-efficacy, physical function, eGFR, and QoL) were analysed using the same SWiM framework. When multiple time points were available, the outcome data closest to intervention completion were prioritised for analysis to ensure consistency in effect estimates.

To address vote counting limitations [24], we supplemented the findings with a narrative synthesis integrating study quality (Risk of bias-2), certainty of evidence assessment (GRADE) and intervention effectiveness trends across different delivery modes and study characteristics [20, 23]. Intervention details were reported using TIDieR guidelines, ensuring standardised reporting of delivery methods, duration, and fidelity [21]. Findings were structured by outcome types (mental health-related, QoL-related or physical health-related outcomes) and effect direction and summary of findings presented in tables for clarity. This SWiM approach, aligned with Cochrane Handbook and PRISMA guidelines, provided a transparent and reproducible synthesis despite study heterogeneity [25].

### Role of funding source

This study is funded by the National Institute for Health and Care Research (NIHR) Maudsley Biomedical Research Centre (BRC). The views expressed are those of the author(s) and not necessarily those of the NHS, the NIHR or the Department of Health and Social Care.

## Results

### Study selection

A total of 500 articles were identified, with 442 unique records remaining after duplicate removal. After title and abstract screening, 45 full-text articles were assessed for eligibility, of which 33 did not meet inclusion criteria, one was unavailable, and one was an ongoing trial. Five studies lacked subgroup data for non-KRT CKD participants; one author provided subgroup data, making the study eligible. Ultimately, five RCTs (N = 631 participants) met full eligibility criteria (Fig. 1).

### Study characteristics

This review included 631 CKD participants from five RCTs [26–30], with sample sizes ranging from 26 to 340 and mean ages between 43.1 (SD = 10.4) and 71.7 (SD = 12.7) years. Most participants were from the UK (58%), followed by Taiwan (18%), China (14%), and the US (10%). Detailed study characteristics are presented in Table 2.

Interventions lasted 4–12 weeks and were delivered digitally [26, 27], via telephone [28], in home-based [29] or combined hospital and digital formats [30]. Controls included usual care, waitlists, or active comparators. Interventions included a range of psychological and behavioural strategies. Greenwood and colleagues delivered Kidney BEAM, a 12-week digital physical activity intervention involving live and on-demand exercise sessions, disease-specific education, and motivational interviewing by specialist renal physiotherapists and patient champions [26]. Gross et al. implemented an 8-week telephone-adapted mindfulness-based stress reduction program featuring teleconference sessions, workshops, and mindfulness practices delivered by certified mindfulness-based stress reduction teachers [28]. Picariello et al. trialled COMPASS, a 12-week therapist-guided digital cognitive behavioural therapy program comprising 11 modules addressing emotional regulation, behaviour change, and stress management, supported by therapist calls who were either trainee or qualified clinical/health psychologists [27]. Tang et al. tested an individualised, home-based exercise program, beginning with hospital-based education delivered by bedside physician and transitioning to a 12-week self-directed exercise routine, emphasising cardiovascular benefits and self-management [29]. Wu et al. introduced a 4-week self-efficacy-based self-management program, combining small group sessions, video content, goal setting, peer learning, and follow-up calls delivered by nurses or case managers [30]. Detailed intervention descriptions as per TiDiER are provided in Supplementary materials Table 3.

**Table 3:** Effects of intervention on primary mental health outcomes of depression and anxiety

Study (Year)	Depression Hedges' g	95% CI	Effect Size Interpretation	95% CI Interpretation	Anxiety Hedges' g	95% CI	Effect Size Interpretation	95% CI Interpretation
Greenwood (2024)	0.04	(− 0.1758, 0.2486)	No effect	❌ Negligible impact with evidence of no effect	0.12	(− 0.0944, 0.3303)	No effect	❌ Negligible impact with evidence of no effect
Gross (2017)	0.72	(0.1569, 1.2748)	Moderate harm	❌ Significant worsening of symptoms with evidence of effect	0.47	(− 0.0831, 1.0147)	Small harm	❓Unclear impact (worsening of symptoms with no clear evidence of effect)
Picariello (2024)	− 0.50	(− 1.2531, 0.2602)	Moderate benefit	❓Unclear impact (symptom reduction with no clear evidence of effect)	− 0.45	(− 1.2718, 0.3649)	Small benefit	❓Unclear impact (symptom reduction with no clear evidence of effect)
Tang (2017)	− 0.68	(− 1.1179, − 0.2456)	Moderate benefit	✅ Significant symptom reductions with evidence of effect	− 0.60	(− 1.0312, − 0.1646)	Moderate benefit	✅ Significant symptom reductions with evidence of effect
Wu (2018)	− 0.66	(− 1.0415, − 0.2717)	Moderate benefit	✅ Significant symptom reductions with evidence of effect	− 0.36	(− 0.7344, 0.0218)	Small benefit	❓Unclear impact (symptom reduction with no clear evidence of effect)

Attrition rates across trials ranged from 6.67% [29] to 21.18% [26], with time constraints and medical reasons commonly cited by authors as the primary reasons for dropout. While all trials applied modified intention-to-treat (Modified-ITT) analyses, handling of missing data varied across studies (See Supplementary materials Table 4).

**Table 4:** Effects of intervention on mental health-related outcomes

Study (Year)	Self-Efficacy Hedges' g	95% CI	Effect Size Interpretation	95% CI Interpretation	Self-Management Hedges' g (95% CI)	95% CI	Effect Size Interpretation	95% CI Interpretation
Greenwood (2024)	N/A	N/A	N/A	N/A	0.24	(0.0237, 0.4495)	Small benefit	✅ Intervention was effective (significant improvements with evidence of effect)
Gross (2017)	N/A	N/A	N/A	N/A	N/A		N/A	N/A
Picariello (2024)	N/A	N/A	N/A	N/A	N/A		N/A	N/A
Tang (2017)	0.87	(0.4239, 1.3111)	Large benefit	✅ Significant improvements in outcome with evidence of effect	N/A		N/A	N/A
Wu (2018)	1.86	(1.4134, 2.3081)	Very Large benefit	✅ Significant improvements in outcome with evidence of effect	2.07	(1.6027, 2.5277)	Very Large benefit	✅ Significant improvements in outcome with evidence of effect

#### Intervention effects

Intervention effects were assessed across primary (depression and anxiety) and secondary (self-efficacy, CKD self-management, QoL, fatigue, physical function, and kidney function) outcomes using Hedges’ g as a standardised measure (Supplementary materials Table 5).

**Table 5:** Effects of intervention on quality-of-life outcomes

Study (Year)	KDQoL (Mental) Hedges' g	95% CI	Effect Size Interpretation	95% CI Interpretation	KDQoL (Physical) Hedges' g	95% CI	Effect Size Interpretation	95% CI Interpretation	HRQoL (EQ-5D-5L) Hedges' g	95% CI	Effect Size Interpretation	95% CI Interpretation
Greenwood (2024)	0.19	(− 0.0206, 0.4059)	Small benefit	❓Unclear effect (improvements with no clear evidence of effect)	− 0.15	(− 0.3679, 0.0582)	No effect	❓Unclear effect (improvements with no clear evidence of effect)	− 0.20	(− 0.4123, 0.0137)	Small harm	❓Unclear effect (worsening of outcomes with no clear evidence of effect)
Gross (2017)	− 0.35	(− 0.9064, 0.1972)	Small harm	❓Unclear effect (improvements with no clear evidence of effect)	− 0.02	(− 0.5654, 0.5294)	No effect	❓Unclear effect (improvements with no clear evidence of effect)	N/A	N/A	N/A	N/A
Picariello (2024)	N/A	N/A	N/A	N/A	N/A	N/A	N/A	N/A	0.28	(-0.5359, 1.087)	Small benefit	❓Unclear effect (worsening of outcomes with no clear evidence of effect)
Tang (2017)	0.34	(− 0.0884, 0.7653)	Small benefit	❓Unclear effect (improvements with no clear evidence of effect)	− 0.60	(− 1.0312, − 0.1646)	Moderate harm	❌ Significant worsening of outcome with evidence of effect	N/A	N/A	N/A	N/A
Wu (2018)	N/A	N/A	N/A	N/A	N/A	N/A	N/A	N/A	N/A	N/A	N/A	N/A

#### Primary mental health outcomes

The effects of interventions on depression and anxiety were mixed (Table 3). Two interventions significantly reduced depression: a self-efficacy-based intervention (Hedges’ *g* = − 0.66, 95% CI [-1.04, -0.27]) [30] and individualised exercise program (Hedges’ *g* = − 0.68, 95% CI [-1.12, -0.25]) [29], both demonstrating moderate effect sizes.

For anxiety, only the individualised exercise program [29] resulted in a moderate, statistically significant reduction (Hedges’ *g* = − 0.60, 95% CI [− 1.03, − 0.16]). The self-efficacy-based intervention [30] showed a small, non-significant effect (Hedges’ *g* = − 0.36, 95% CI [− 0.73, 0.02]).

COMPASS using digital cognitive behavioural therapy showed moderate-to-small effects on both depression (Hedges’ g = -0.50, 95% CI [− 1.25, 0.26]) and anxiety (Hedges’ *g* = − 0.45, 95% CI [− 1.27, 0.36]) [27]. However, confidence intervals included zero, indicating statistical uncertainty, likely due to the small CKD subgroup (*N* = 26) analysed in this review.

In contrast, telephone-adapted mindfulness-based stress reduction significantly worsened depressive symptoms (Hedges’ *g* = 0.72, 95% CI [0.16, 1.27]), while its effect on anxiety was small and statistically uncertain with a wide confidence interval (Hedges’ *g* = 0.47, 95% CI [-0.08, 1.01]) [28]. Kidney BEAM, a digital physical activity-based intervention, showed no significant effects on either depression (Hedges’ *g* = 0.04, 95% CI [− 0.18, 0.25]) or anxiety (Hedges’ *g* = 0.12, 95% CI [− 0.09, 0.33]) symptoms [26].

#### Mental health-related outcomes

Mental health-related outcome effects are summarised in Table 4. Self-efficacy improved significantly, with large-to-very large effect sizes. Self-efficacy-based intervention demonstrated a substantial impact (Hedges’ *g* = 1.86, 95% CI [1.41, 2.31]) [30], while Tang et al. [29] also reported a large effect post-intervention (Hedges’ g = 0.87, 95% CI [0.42, 1.31]) on self-efficacy after individualised exercise program*.*

For self-management, self-efficacy-based intervention demonstrated a very large effect (Hedges’ *g* = 2.07, 95% CI [1.60, 2.53]) [30], while Kidney BEAM showed a small but significant effect (Hedges’ *g* = 0.24, 95% CI [0.02, 0.45]) [26]. No other studies assessed these outcomes.

#### Quality of life (QoL)

QoL findings were inconsistent (Table 5). For mental-health component of KDQoL, both physical activity-based interventions, i.e., individualised exercise program (Hedges’ *g* = 0.34, 95% CI [− 0.09, 0.77]) [29] and Kidney BEAM (Hedges’ *g* = 0.19, 95% CI [-0.02, 0.41]) [26] showed small, non-significant improvements. Telephone-adapted mindfulness-based stress reduction showed a small, non-significant decline (Hedges’ *g* = − 0.35, 95% CI [− 0.91, 0.20]) in KDQoL (Mental) [28].

For physical-health component of KDQoL, individualised exercise program led to a moderate but significant decline (Hedges’ *g* = − 0.60, 95% CI [− 1.03, − 0.16]) [29]. However, Kidney BEAM (Hedges’ *g* = − 0.15, 95% CI [− 0.37, 0.06]) [26] and telephone-adapted mindfulness-based stress reduction Hedges’ *g* = − 0.02, 95% CI [− 0.57, 0.53]) [28] showed no significant effects.

For overall QoL (EQ-5D-5L), COMPASS showed a small, non-significant benefit (Hedges’ *g* = 0.28, 95% CI [− 0.54, 1.09]) [27], while Kidney BEAM resulted in a small, non-significant decline (Hedges’ *g* = − 0.20, 95% CI [− 0.41, 0.01]) [26].

#### Physical health-related outcomes

Physical health-related outcome effects were variable (Table 6). Fatigue levels remained unchanged post-intervention in studies that measured this outcome. Kidney BEAM reported no significant post-intervention effect (Hedges’ *g* = 0.02, 95% CI [− 0.20, 0.23]) [26] whereas telephone-adapted mindfulness-based stress reduction showed a very small but non-significant improvement (Hedges’ *g* = 0.15, 95% CI [− 0.40, 0.70]) [28].

**Table 6:** Effects of intervention on physical health-related outcomes

Study (Year)	Fatigue Hedges' g	95% CI	Effect Size Interpretation	95% CI Interpretation	Physical Function Hedges’ g	95% CI	Effect Size Interpretation	95% CI Interpretation	CKD clinical markers (eGFR) Hedges' g	95% CI	Effect Size Interpretation	95% CI Interpretation
Greenwood (2024)	0.02	(− 0.1967, 0.2276)	No effect	❌ Negligible impact with evidence of no effect	0.28	(0.0652, 0.4915)	Small benefit	✅ Significant improvements with evidence of effect	0.00	(− 0.2177, 0.2177)	No effect	❌ Negligible impact with evidence of no effect
Gross (2017)	0.15	(− 0.4028, 0.7028)	No effect	❓Unclear effect (improvements with no clear evidence of effect)	N/A	N/A	N/A	N/A	N/A	N/A	N/A	N/A
Picariello (2024)	N/A	N/A	N/A	N/A	N/A	N/A	N/A	N/A	N/A	N/A	N/A	N/A
Tang (2017)	N/A	N/A	N/A	N/A	− 0.62	(1.055, 0.1869)	Moderate benefit	✅ Significant improvements with evidence of effect	N/A	N/A	N/A	N/A
Wu (2018)	N/A	N/A	N/A	N/A	N/A	N/A	N/A	0.07	(− 0.3032, 0.4474)	N/A	No effect	❌ Negligible impact with evidence of no effect

Physical function improved significantly in both interventions that assessed this outcome. Kidney BEAM showed a small, significant improvement (Hedges’ *g* = 0.28, 95% CI [0.07, 0.49]) [26] whereas individualised exercise program led to a moderate, significant improvement (Hedges’ *g* = 0.62, 95% CI [1.06, 0.19]) [29] in physical function assessed using the Sit-to-Stand (STS) tests.

No changes were reported in the CKD clinical marker eGFR by Kidney BEAM (Hedges’ *g* = 0.00, 95% CI [− 0.22, 0.22]) [26] or self-efficacy-based intervention (Hedges’ *g* = 0.07, 95% CI [− 0.30, 0.45]) [30]. Both studies assessing this outcome reported negligible effects, with confidence intervals crossing zero highlighting that psychological and behavioural interventions may not influence clinical outcomes in the short-term.

#### Synthesis using vote counting based on direction of effects

**Fig. 2:**
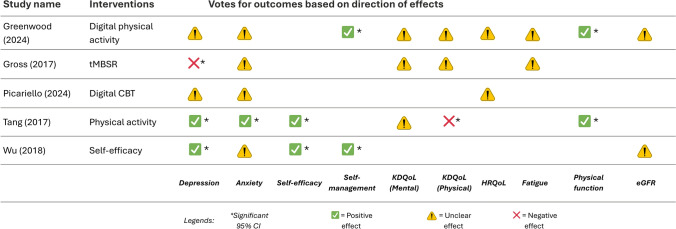
Synthesis of outcomes using vote counting based on direction of effects

Given the heterogeneity of interventions and outcome measures, vote counting was used to synthesise intervention effectiveness by summarising the direction of effects (positive, unclear, or negative) rather than relying solely on statistical significance [25] as summarised in Fig. 2.

#### Mental health-related outcomes

For depression, 40% of studies showed a positive effect [29, 30], 20% showed a negative effect [28], and 40% showed unclear effect [26, 27]. For anxiety, 80% of studies showed an unclear effect, with only 20% demonstrating a positive effect [29]. Self-efficacy and self-management improved in 100% of studies that measured this outcome, with large-to-very large positive effects, indicating compelling evidence of benefit [29, 30].

#### Quality of life (QoL)

For KDQoL (Mental) and overall QoL (EQ-5D-5L), 100% of studies that reported these outcomes showed unclear effects. For KDQoL (Physical), 33% showed a negative effect [29], while 67% showed unclear effect [26, 28].

#### Physical health-related outcomes

Physical function consistently showed a positive effect in 100% of studies (2 out of 2), demonstrating strong evidence of benefit [26, 29]. Fatigue and kidney function (eGFR) showed no effect in 100% of studies (2 out of 2), indicating no evidence that psycho-behavioural interventions had an impact.

### Risk of bias assessment

**Fig. 3:**
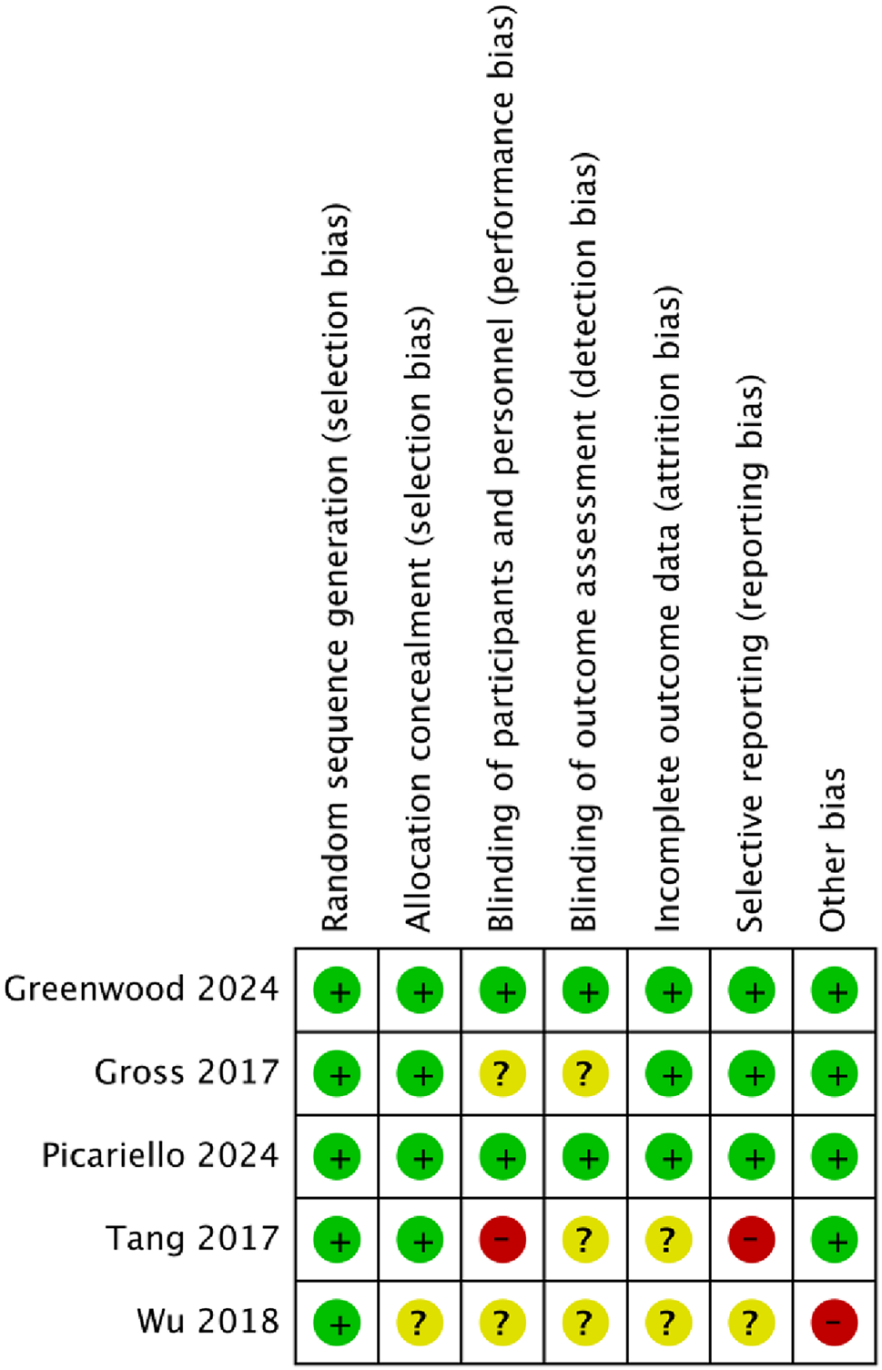
Risk of bias (RoB-2) assessment of included studies

Risk of bias was assessed using the Risk of bias-2 tool, evaluating selection, performance, detection, attrition, and reporting biases across studies (Fig. 3).

Random sequence generation was rated low risk in all studies while allocation concealment was adequate in most cases, except for Wu (2018), which was rated unclear due to insufficient reporting. Performance bias was a concern in Tang (2017; high risk) and Gross (2017) and Wu (2018; unclear risk), where lack of blinding may have influenced self-reported outcomes such as depression, anxiety, and QoL. Detection bias was generally low, except in Tang (2017) and Wu (2018) where it was unclear, thus raising concerns about potential assessor bias. Attrition bias was unclear in Tang (2017) and Wu (2018) due to incomplete outcome data and insufficient reporting on missing data. Selective reporting bias was high risk in Tang (2017) and unclear in Wu (2018), reflecting potential deviations from pre-specified outcomes. Greenwood (2024) deviated slightly from the pre-registered protocol and did not report 'frailty score’. Given this was neither a primary/secondary outcome of this review; bias in selective outcome reporting was not flagged.

**Fig. 4:**
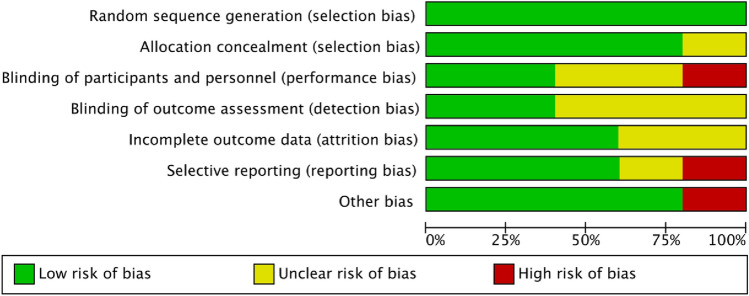
Overall distribution of bias across domains

Overall, Greenwood (2024) and Picariello & Hulme (2024) had the lowest risk of bias, increasing confidence in their findings, while Tang (2017) and Wu (2018) showed higher risk, particularly in blinding and reporting domains, potentially affecting reliability. Overall distribution of risk across domains is depicted in Fig. 4.

### Certainty of evidence

Certainty of evidence, assessed using the GRADE approach, varied across ten outcomes from five RCTs (*N* = 631) and is summarised in Table 7. Full GRADE assessment is attached as Supplementary materials Table 6.

**Table 7:** Summary of certainty of evidence (GRADE) for all outcomes

Outcome	Overall Certainty	Key Findings
Depression	⊕ ⊕ ◯◯ (Low)	Significant symptom reductions in two studies, but bias and imprecision lowered certainty
Anxiety	⊕ ⊕ ◯◯ (Low)	One study showed improvement; 80% had unclear effects with large CIs indicating serious imprecision
Self-Efficacy	⊕ ⊕ ⊕ ◯ (Moderate)	All studies showed large, consistent effects. Small sample size lowered certainty
Self-Management	⊕ ⊕ ◯◯ (Low)	All studies showed improvement, but bias in Wu (2018) and small sample size lowered certainty
KDQoL (Mental)	⊕ ⊕ ◯◯ (Low)	All studies reported unclear or non-significant effects, with bias concerns
KDQoL (Physical)	⊕ ⊕ ◯◯ (Low)	Mixed results: Tang (2017) showed worsening; others showed no effect
Overall QoL (EQ-5D-5L)	⊕ ◯◯◯ (Very Low)	Unclear effects, with concerns about imprecision and inconsistency. Small sample size lowered certainty
Fatigue	⊕ ◯◯◯ (Very Low)	No effect or unclear results, with high imprecision and small sample size
Physical Function (Sit-to-Stand test)	⊕ ⊕ ⊕ ◯ (Moderate)	Significant, consistent improvements across studies. Small sample size lowered certainty
Kidney Function (eGFR)	⊕ ◯◯◯ (Very Low)	No significant effects, with high bias and imprecision. Small sample size lowered certainty

Moderate-certainty evidence was found for self-efficacy (*N* = 366) and physical function (*N* = 397), both demonstrating consistent, large effects across studies. However, small sample sizes led to downgrading due to imprecision.

Low-certainty evidence supported outcomes effects of depression (*N* = 631), anxiety (*N* = 631), self-management (N = 366), and QoL (*N* = 518–397). Depression outcomes were mixed, with two studies showing significant symptom reductions while one study (Gross, 2017) indicated worsening symptoms, contributing to serious imprecision.

Anxiety findings were inconclusive, with only one study showing improvement, while 80% of studies reported unclear or non-significant effects with large confidence intervals. Self-management showed positive effects across all studies, though bias in Wu (2018) and small sample sizes lowered certainty. QoL outcomes (mental and physical) were highly variable, with some studies showing improvement, while others, particularly Tang (2017), reported worsening effects, leading to low confidence in findings.

Very-low-certainty evidence was found for overall QoL (EQ-5D-5L), fatigue (*N* = 397), and kidney function (eGFR, *N* = 397) due to serious imprecision, small sample sizes, and inconsistent effects. Fatigue outcomes were unclear, while kidney function remained unchanged across interventions, suggesting psycho-behavioural interventions do not directly impact kidney function over short follow-up periods.

Overall, self-efficacy and physical function showed the most consistent benefits, reinforcing the potential role of psycho-behavioural interventions in improving patient engagement and mobility in CKD. Evidence for depression and self-management were promising although low certainty, while effects on anxiety, QoL and clinical markers remain uncertain, emphasising the need for larger, well-powered RCTs with longer follow-up to determine sustained effects and optimise integration into CKD care pathways.

## Discussion

This systematic review is the first to synthesise evidence from RCTs assessing psychological and behavioural interventions specifically in CKD, addressing a critical gap where research has predominantly focused on kidney failure populations [16, 17]. We identified moderate-certainty evidence supporting improvements in self-efficacy and physical function and low-certainty evidence for depression and self-management. Effects on anxiety, QoL, and clinical markers, however, remain inconclusive due to methodological limitations, intervention heterogeneity, and imprecision.

In line with broader literature [31, 32], this review demonstrated that structured behavioural activation via self-efficacy and self-management training [30] and physical activity [29] improved depressive symptoms, reinforcing their potential in enhancing psychological resilience in CKD. However, effects on anxiety were inconsistent, with only one study [29] reporting significant reductions associated with their individualised exercise program. This aligns with existing evidence linking physical activity to neurobiological and behavioural pathways that mitigate anxiety [33].

Notably, COMPASS, a cognitive behavioural therapy-based intervention [27] yielded non-significant effects on depression and anxiety, diverging from previous meta-analyses [16, 34] in kidney failure demonstrating benefits associated with cognitive behavioural therapy-based psychotherapies. This discrepancy is likely attributable to limited statistical power due to the small CKD subgroup (*N* = 26), rather than true intervention inefficacy. Importantly, the intervention was developed for individuals with long-term conditions more broadly and was not tailored to the specific psychological needs of CKD patients, who are known to experience elevated rates of depression and anxiety relative to other chronic disease populations [3, 4]. Furthermore, the highest dropout rates in the trial were observed among CKD participants, suggesting that non-specific interventions may be less engaging or effective in this group. These findings underscore the need for adequately powered trials of CKD-specific psychological interventions.

Contrary to expectations, telephone-adapted mindfulness-based stress reduction [28] worsened depressive symptoms, raising questions about the suitability of mindfulness-based strategies in CKD. Disease-specific stressors, fatigue, symptom burden, medical uncertainty, may necessitate active coping mechanisms rather than passive acceptance strategies. This result contradicts findings in solid organ transplant, dialysis patients and other clinical populations, where mindfulness-based stress reduction demonstrated sustained psychological benefits [35–37] on symptoms of depression and anxiety. Possible explanations include higher baseline anxiety in the intervention group, as well as non-specific therapeutic effects such as therapist attention and group support which are known to account for up to 30% of patient improvement in unblinded mental health trials [38]. Additionally, the shortened intervention dose to minimise patient burden may have attenuated the therapeutic effects of telephone-adapted mindfulness-based stress reduction. Future trials should investigate whether increased intervention dosage and rigorous randomisation strategies reduce these anomalies.

QoL outcomes were heterogeneous. Small improvements in mental health-related QoL were observed following physical activity interventions [26, 29], while telephone-adapted mindfulness-based stress reduction [28] showed declines. These findings contradict recent RCTs demonstrating the positive effects of mindfulness-based stress reduction on perceived QoL in kidney failure cohorts [39], underscoring the possibility of trial-specific confounders rather than intervention inefficacy. Similarly, physical health-related QoL worsened after individualised exercise intervention [29], diverging from the positive effects reported in dialysis populations [16]. Heightened symptom awareness or exercise-related discomfort may underlie this perceived decline.

Additionally, both digital interventions COMPASS [27] and Kidney BEAM [26] had minimal impact on overall QoL (EQ-5D-5L). This suggests a need for more tailored, patient-centred digital interventions to address CKD patients' unique psychosocial needs.

Consistent improvements in physical function provide moderate-certainty support for structured physical activity, aligning with evidence that exercise preserves mobility, reduces frailty, and enhances functional independence in CKD [40]. Given the progressive trajectory of CKD, early interventions to maintain functional capacity are critical.

Conversely, fatigue remained unchanged, reflecting its multifactorial nature involving inflammation, anaemia, and uraemic toxins: factors unlikely to be fully mitigated by short-term behavioural interventions [41, 42]. Similarly, no improvements were observed in CKD clinical markers (eGFR). While depression and anxiety are associated with renal decline [5], the short duration (4–12 weeks) of included interventions potentially precluded measurable impacts. Nonetheless, improvements in self-efficacy, self-management, and physical activity may enhance long-term adherence to CKD care, meriting evaluation in longer-duration trials.

A key limitation of our study is the small number of eligible trials and participants, reflecting an under-researched population despite evident psychosocial need. This restricts the generalisability and robustness of findings, highlighting the need for expanded research focus on CKD populations. Although RCTs were selected to maximise methodological rigour, the risk of bias remained moderate-to-high, particularly in blinding [29, 30] and selective reporting [28]. While blinding is inherently challenging in psychological interventions, lack thereof introduces performance and detection bias, limiting certainty. Further, intervention heterogeneity, delivery modalities, and varied outcome assessments precluded meta-analysis. In accordance with PRISMA and Cochrane guidance, vote counting was employed [25]. However, standardised effect sizes (Hedges’ g) may inadequately capture clinically meaningful changes. Mean Adjusted Differences (MAD) provide more clinically interpretable insights, as demonstrated in KDQoL-Mental in Kidney BEAM trial [26], where a small, standardised effect masked a meaningful absolute change (3 Arbitrary Units) between groups. Finally, short follow-up durations limited conclusions on long-term efficacy, particularly on clinical markers. Trials with extended follow-up are essential to evaluate sustained psychological benefits and clinical outcomes.

This review underscores the importance of embedding psychological and behavioural interventions early in kidney care pathways. Moderate-certainty evidence supports integrating structured self-management and tailored exercise to improve patient engagement and physical function. However, inconclusive evidence on depression, anxiety and QoL highlights a pressing need for CKD-specific psychological interventions. Moreover, the absence of effect on fatigue and disease markers suggests that psycho-behavioural interventions may exert benefits indirectly, via sustained self-care behaviours.

Future research should prioritise well-powered, CKD-focused RCTs with longer follow-up durations. These studies must ensure balanced gender representation and include participants from diverse socio-economic and ethnic backgrounds to improve the generalisability of findings. Further trials are needed to evaluate face-to-face interventions in CKD, enabling comparisons across delivery formats. Multi-component interventions incorporating self-management, cognitive restructuring, and personalised exercise may yield enhanced efficacy. Qualitative research exploring patient perspectives is needed to optimise intervention acceptability, alongside the adoption of standardised outcomes to facilitate cross-trial comparability.

## Conclusion

This review demonstrates consistent benefits of self-efficacy and physical activity interventions for improving self-management and physical function in CKD. While low-certainty evidence suggests benefits for depression and self-management, uncertainty persists regarding anxiety, QoL, and kidney outcomes. Addressing the unique psychosocial challenges in CKD will require well-designed, multi-component interventions tested over longer durations and integrated systematically into kidney care pathways to maximise patient-centred outcomes.

## Data Availability

All data generated or analysed during this study are included in this published article and its supplementary information files.

## Abbreviations

ACT: Acceptance and commitment therapy
AU: Arbitrary units
BDI: Beck depression inventory
BRC: Biomedical research centre
CBT: Cognitive behavioural therapy
CI: Confidence interval
CKD: Chronic kidney disease
eGFR: Estimated glomerular filtration rate
EQ-5D-5L: EuroQol 5-dimensions 5-levels questionnaire
ESKD: End-stage kidney disease
GRADE: Grading of recommendations assessment, development and evaluation
HRQoL: Health-related quality of life
HPA axis: Hypothalamic–pituitary–adrenal axis
ITT: Intention-to-treat
KDIGO: Kidney disease: improving global outcomes
KDQoL: Kidney disease quality of life questionnaire
KDOQI: Kidney disease outcomes quality initiative
KRT: Kidney replacement therapy
LTC: Long-term condition
MAD: Mean adjusted difference
MBCT: Mindfulness-based cognitive therapy
MBSR/t-MBSR: Mindfulness-based stress reduction/telephone-adapted mindfulness-based stress reduction
NHS: National health service
NIHR: National institute for health and care research
PICOS: Population, intervention, comparator, outcomes, study design
PRISMA: Preferred reporting items for systematic reviews and meta-analyses
PROSPERO: International prospective register of systematic reviews
QoL: Quality of life
RCT: Randomised controlled trial
RoB-2: Cochrane risk of bias tool for RCTs
SD: Standard deviation
STS test: Sit-to-stand test
SWiM: Synthesis without meta-analysis
TIDieR: Template for intervention description and replication
